# Probiotic and Synbiotic Interventions Targeting Oxalate-Degrading Gut Bacteria for the Prevention of Kidney Stones: A Systematic Review

**DOI:** 10.7759/cureus.98728

**Published:** 2025-12-08

**Authors:** Maanya Bhardwaj, Abhinav Singhal, Gaurika Bhardwaj, Ivo Dukic

**Affiliations:** 1 Urology, Cambridge University Hospitals NHS Foundation Trust, Cambridge, GBR; 2 Urology, University Hospitals Birmingham NHS Foundation Trust, Birmingham, GBR; 3 Surgery, Royal Brompton and Harefield NHS Foundation Trust, London, GBR

**Keywords:** bifidobacterium, kidney stone, lactobacillus, nephrolithiasis, oxalate-degrading bacteria, oxalobacter formigenes, probiotic agent, synbiotic agent, urinary stone, urolithiasis

## Abstract

Kidney stone disease (KSD) is a common and recurrent healthcare problem. Calcium oxalate stones are the most common type of stones, influenced by urinary oxalate levels. Increasingly, the role of the gut microbiome is being studied, particularly oxalate-degrading bacteria such as *Oxalobacter formigenes*, *Lactobacillus*, and *Bifidobacterium*, as potential therapeutic targets. Probiotic and synbiotic interventions aimed at enhancing intestinal oxalate degradation have, therefore, been proposed as strategies to reduce urinary oxalate excretion and mitigate stone recurrence.

A comprehensive search of PUBMED, Cochrane Library, Scopus, and Embase databases was conducted following the Preferred Reporting Items for Systematic Reviews and Meta-Analyses (PRISMA) guidelines. A total of nine studies, published between January 1, 2000, and September 3, 2025, met the inclusion criteria, comprising five randomized controlled trials and four observational studies. Interventions included *O. formigenes* preparations, lactic acid bacteria mixtures, and multi-strain probiotics or synbiotics. Urinary oxalate excretion was the most consistently reported outcome, while assessment of kidney stone recurrence was limited due to short study durations and follow-ups.

Across trials, probiotic or synbiotic therapy did not consistently reduce urinary oxalate levels compared with placebo or standard care. Although several studies demonstrated successful gastrointestinal colonization with *O. formigenes*, this did not translate into meaningful biochemical improvements. One randomized trial reported a reduction in the incidence of hyperoxaluria compared with an active comparator; however, this finding was not replicated elsewhere, and no included study demonstrated a significant reduction in clinically confirmed stone recurrence. Overall, the quality of evidence was limited by small sample sizes, heterogeneity in probiotic strains, dosing regimens, and short intervention periods.

Current evidence does not support the use of probiotics or synbiotics targeting oxalate-degrading bacteria as effective therapies for reducing urinary oxalate excretion or preventing kidney stone recurrence. Despite their biological plausibility and favourable safety profile, their clinical utility remains unproven. Future research should prioritize long-term, randomized trials using standardized microbial formulations and incorporating imaging-confirmed recurrence outcomes to more definitively establish the role of microbiome-based interventions in KSD prevention.

## Introduction and background

Kidney stone disease (KSD) affects 15% of the world’s population, causing considerable physical, psychological, and financial burden [1]. Recurrence rates are high, with up to 50% of individuals who have had a stone experiencing a second stone within 10 years [2]. KSD has a detrimental impact on a patient’s quality of life, as kidney stones can lead to recurrent episodes of acute pain, chronic pain, obstructive uropathy, renal failure, urinary tract infections, haematuria, and the need for surgical intervention [3]. 

Kidney stones are chemically diverse, with calcium-containing stones being the most prevalent [4]. Around 80% of these are composed of calcium oxalate [5]. Among various contributing factors, dietary oxalate plays a central role in determining urinary oxalate levels [5,6]. Hyperoxaluria, or elevated oxalate in the urine, is the most significant risk factor for calcium oxalate stone formation [6]. The oxalate-to-calcium molar ratio, typically maintained at 1:10, is crucial, as even slight increases in urinary oxalate are more likely to trigger crystallization than changes in calcium levels [6]. Humans cannot metabolize oxalate, which is toxic, and must rely on excretion through urine or faeces as insoluble calcium oxalate, or microbial degradation in the gut [7]. The gut microbiome, particularly oxalate-degrading bacteria like *Oxalobacter formigenes*, has emerged as a potential modulator of urinary oxalate levels [7]. This has led to interest in probiotic and synbiotic interventions aimed at enhancing oxalate degradation in the gastrointestinal tract. Although these approaches show promise in reducing calcium oxalate stone formation, current evidence remains inconsistent. No comprehensive synthesis of interventional studies has yet been conducted, leaving the clinical effectiveness of such microbiome-targeted therapies uncertain. 

## Review

Methods

This systematic review was conducted in accordance with the Preferred Reporting Items for Systematic Reviews and Meta-Analyses (PRISMA) guidelines [8]. The study protocol was registered on the PROSPERO website, and the ID is CRD420251139899. Studies were selected in accordance with the inclusion and exclusion criteria outlined in Table 1.

**Table 1:** Overview of the inclusion and exclusion criteria for selection of studies

	Inclusion	Exclusion
Population	Adult patients with history of kidney stones, paediatric patients with a history of kidney stones, includes patients who are recurrent stone formers	Pregnant patients, animal or in vitro studies
Intervention	Probiotic interventions containing oxalate-degrading bacteria (e.g., *Oxalobacter formigenes*, *Lactobacillus*, *Bifidobacterium*) and synbiotic interventions (probiotic + prebiotic combinations) designed to enhance oxalate-degrading activity	-
Comparator	Placebo, no treatment/standard care (hydration, dietary advice, citrate supplementation), alternative probiotic or synbiotic strains/formulations	-
Study design	Randomized controlled trials, prospective or retrospective cohort studies, case series, case control studies	Case reports, editorials, conference abstracts without extractable data, systematic reviews and meta-analysis, pilot interventions
Language	Papers in English	Non-English studies
Time Frame	Publications between January 1, 2000 and September 3, 2025	Studies not conducted in this timeframe
Outcomes	Studies reporting at least one primary or secondary outcome - Primary Outcomes: Urinary oxalate excretion, Kidney stone recurrence (new or recurrent stones confirmed clinically or via imaging); Secondary Outcomes: Other urinary biochemical parameters (calcium, citrate, uric acid, pH), Colonization or persistence of administered bacterial strains in the gut, Patient-reported outcomes	Studies not reporting on relevant clinical outcomes

**Figure 1:**
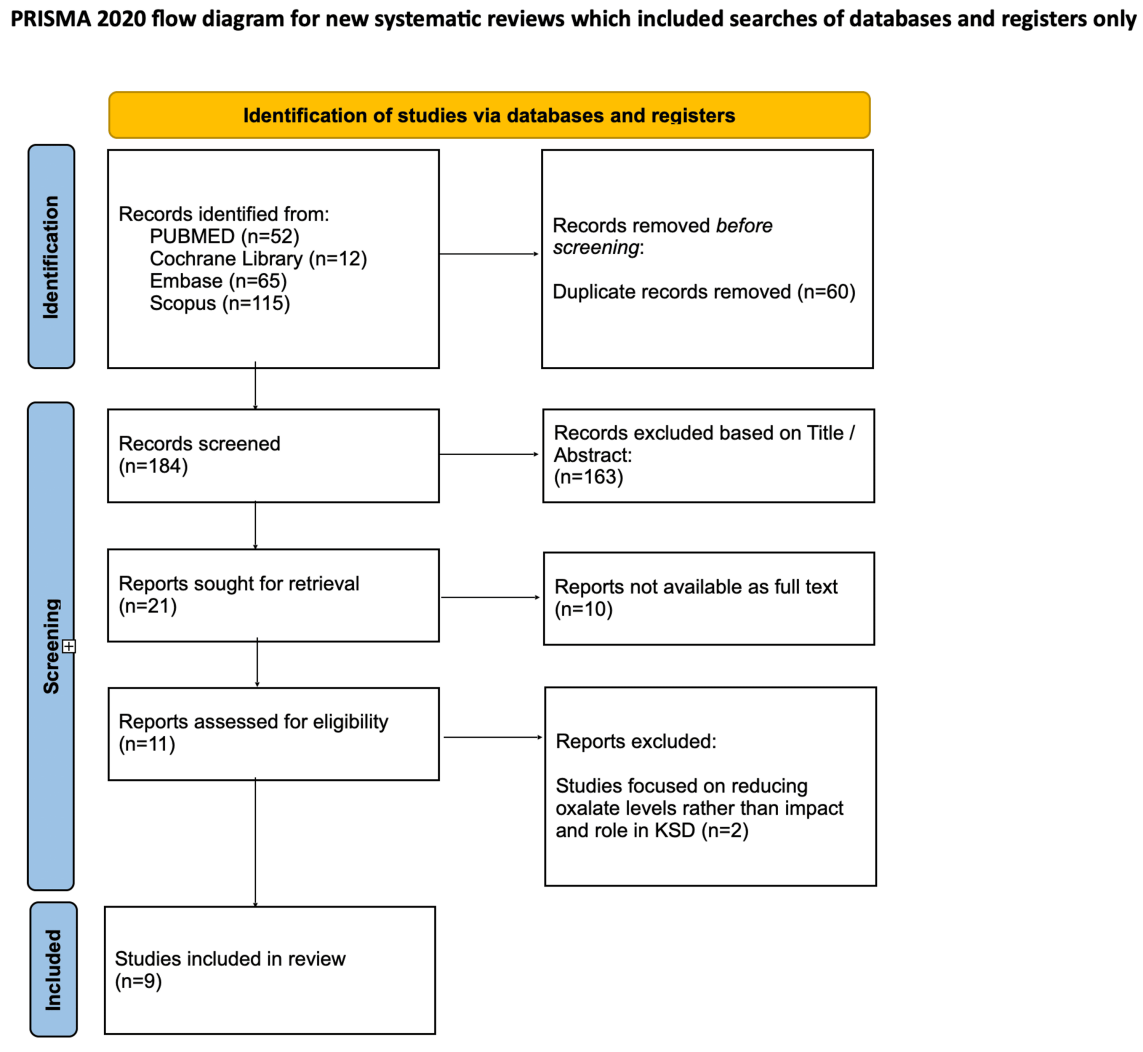
PRISMA flow diagram for selecting studies for inclusion in the review PRISMA: Preferred Reporting Items for Systematic Reviews and Meta-Analyses

A comprehensive search was conducted using electronic databases, including PubMed, MEDLINE, PMC, Cochrane, EMBASE, and SCOPUS. Articles published from January 1, 2000, to September 3, 2025, were included in the search. A structured search strategy was developed using keywords and Medical Subject Headings (MeSH) terms. The search terms included combinations of urolithiasis, nephrolithiasis, kidney calculus, kidney stone, renal stone, urinary stone, probiotic agent, synbiotic agent, prebiotic agent, *O. formigenes*, *Lactobacillus*, *Bifidobacterium*, and oxalate-degrading bacteria. Search strategies used to identify relevant papers in the databases are available in Appendix A (Table 6). Titles and abstracts were screened independently by three reviewers (AS, MB, and GB) using the defined inclusion and exclusion criteria in Table 1. Full texts for potentially eligible studies were retrieved and assessed for final inclusion. The PRISMA flow diagram in Figure 1 provides an overview of the process used for study selection [8]. Data were extracted independently by two reviewers using a standardized data extraction form. The data collected included study characteristics, population details, intervention details, outcome measures, key findings, and conclusions. The methodological quality of included studies was assessed using the Cochrane Risk of Bias 2.0 [9] tool for randomized controlled trials (RCTs) and Risk of Bias in Non-randomized Studies - of Interventions-I (ROBINS-I) [10] for non-randomized interventional studies. Discrepancies among the three reviewers were addressed through discussion and resolved by consensus after reviewing the inclusion and exclusion criteria.

A meta-analysis was not performed due to substantial heterogeneity across the included studies in terms of study design, patient populations, probiotic and synbiotic formulations, dosing regimens, treatment durations, follow-up durations, and outcome reporting. The interventions varied widely, ranging from *O. formigenes* preparations to multi-strain lactic acid bacterial products, with inconsistent strain viability and colonization assessments. Outcome measures were similarly variable, with differences in how urinary oxalate was quantified, whether dietary intake was controlled, and whether secondary metabolic markers were reported. Several studies lacked appropriate control groups or had insufficiently reported data, further limiting comparability. Collectively, these methodological inconsistencies precluded the pooling of results and prevented the generation of a reliable or clinically meaningful meta-analytic estimate. 

Results

The database search identified a total of 244 records. After removal of duplicates, 184 titles and abstracts were screened, of which 11 full-text articles were assessed for eligibility. A total of nine studies eventually met the inclusion criteria and were included in the qualitative synthesis. Of these, five were RCTs and four were interventional or observational studies. Table 2 outlines the key characteristics of the included studies. 

**Table 2:** Summary of key characteristics of included studies CaOX: Calcium oxalate; CFU: Colony forming units; Ca: Calcium; GI: Gastrointestinal; AE: Adverse effects; RCT: Randomized controlled trial; LAB: Lactic acid bacteria; Mg: Magnesium; Na: Sodium; K: Potassium; KMgCit: Potassium magnesium citrate; SS: Super saturation

Author	Design	Population	Intervention	Comparator	Primary outcomes	Secondary outcomes	Main findings	Adverse events
Campieri et al. [11]	Interventional, within-subject	10 CaOx stone formers with hyperoxaluria	Lactic acid bacteria mix (*Lactobacillus acidophilus*, *Streptococcus thermophilus*, *Lactobacillus bulgaricus*, etc., ~10⁹ CFU daily × 4 wks)	Baseline	24-h urinary oxalate	Urinary Ca, citrate; stool counts	~44% reduction in urinary oxalate; minor changes in other markers	Mild GI discomfort; no serious AE
Goldfarb et al. [12]	RCT, double-blind, placebo-controlled	20 CaOx stone formers with idiopathic hyperoxaluria	“Oxadrop” (LAB mix, 3.6 g/day × 28 days)	Placebo	24-h urinary oxalate	Wash-out effects at 56 d; urinary profile	No significant oxalate reduction vs placebo	Mild constipation in 3; otherwise safe
Ferraz et al. [13]	Within-subject intervention, dietary	14 stone formers (no hyperoxaluria)	*Lacticaseibacillus casei *+ *Bifidobacterium breve* (lyophilized, t.i.d. × 2 wks) during oxalate-rich diet (200 mg/d)	High-oxalate diet alone	24-h urinary oxalate	Urine Ca, Mg, citrate, Na, K	Modest oxalate decrease; not statistically significant overall; variable individual response	Well tolerated
Jairath et al. [14]	RCT	80 patients with hyperoxaluria	*Oxalobacter formigenes *(7 × 10⁸ organisms bid × 1 mo)	Potassium-Mg citrate (30 mEq bid)	Incidence of hyperoxaluria	Urine metabolic markers; serum labs	Probiotic markedly reduced hyperoxaluria vs KMgCit (82.5%→15% vs 77.5%→37.5%)	Mild symptoms in comparator group; probiotic well-tolerated
Hoppe et al. [15]	Pilot RCT/interventional	13 patients (various kidney function levels)	*O. formigenes* (IxOC-2 strain; oral)	Baseline/no treatment	Plasma oxalate; urinary oxalate	Safety	Plasma oxalate reduced in 10/13; urinary oxalate fell 22–48% in 3/5 with normal renal function	No serious AE
Hoppe et al. [16]	Phase I/II RCT, double-blind	28 (14 active, 14 placebo)	*O. formigenes* (Oxabact® OC5, ≥10⁹ CFU twice daily × 8 wks)	Placebo	24-h urinary oxalate	Plasma oxalate; stool colonization	No significant change; colonization successful	Mild GI events; one pyelonephritis
Lieske et al. [17]	RCT, double-blind, placebo-controlled	40 CaOx stone formers with mild hyperoxaluria	Oxadrop (LAB mix, 2 g/day) or Synbiotic AKSB (multi-strain + prebiotic) × 4 wks under controlled diet	Placebo	24-h urinary oxalate	Urinary Ca, citrate, CaOx SS; stool flora	Controlled diet ↓ oxalate; probiotics/synbiotics gave no extra benefit	Mild GI; safe
Lieske et al. [18]	Cross-sectional + small intervention	~247 stone formers + controls; subset given Oxadrop	Oxadrop LAB probiotic (~10⁹-10¹¹ CFU)	Baseline	*O. formigenes* colonization; urinary oxalate	Stone risk	Colonization linked to lower urinary oxalate; probiotics failed to colonize effectively	Not specified
Tavasoli et al. [19]	RCT, double-blind, placebo-controlled	100 randomized (~50/arm)	Probiotic mix (*L. acidophilus*, *B.* *lactis*, *B. bifidum*, *B. longum*; 1.8 × 10⁹ CFU/cap, 2 capsules/day × 4 wks)	Placebo	24-h urinary oxalate	Urine Ca, citrate, uric acid, pH, volume; stool colonization	No difference vs placebo; colonization largely unsuccessful	Well tolerated

The included studies were published between 2000 and 2025 and involved sample sizes ranging from 6 to 100 participants. Interventions included *O. formigenes* preparations (Oxabact® or other formulations) in five trials, and lactic acid bacteria mixtures (e.g., Oxadrop®, Campieri’s freeze-dried probiotic cocktail) in four trials. Three trials involved other probiotics or synbiotics, such as *Lactobacillus casei* + *Bifidobacterium breve*, synbiotic Agri-King Synbiotic, or multi-strain preparations. 

Populations varied among the studies, including recurrent calcium oxalate stone formers, idiopathic hyperoxaluria patients, and stone formers without hyperoxaluria. Intervention durations ranged from 4 weeks to 24 weeks. All studies reported urinary oxalate excretion as a primary outcome; secondary outcomes included urinary supersaturation indices, colonization of the administered strains, plasma oxalate, tolerability, and safety. 

Effects on Urinary Oxalate Excretion 

Across RCTs, no consistent evidence of a significant reduction in urinary oxalate excretion was observed with probiotics or *O. formigenes* compared with placebo. Hoppe et al. [16] showed successful gut colonization, but no significant reduction in urinary or plasma oxalate. Tavasoli et al. [19] and Goldfarb et al. [12] similarly reported no significant differences compared with placebo. Jairath et al. [14] were an exception, reporting a marked reduction in the proportion of patients with hyperoxaluria after one month of *O. formigenes* therapy compared with potassium-magnesium citrate. 

In within-subject interventional studies, such as Campieri et al. [11] and Ferraz et al. [13], modest reductions in urinary oxalate were noted in some participants, but effects were inconsistent and often not statistically significant. 

Stone Recurrence, Metabolic, and Microbiological Outcomes 

None of the included RCTs had sufficient duration or power to assess kidney stone recurrence as a primary outcome. No consistent effect on stone recurrence was demonstrated. Several studies evaluated urinary supersaturation and biochemical markers. No consistent improvements in urinary calcium, citrate, or supersaturation indices were observed across studies. Plasma oxalate was reduced in a subset of patients with normal renal function treated with *O. formigenes* in early pilot trials, but this was not confirmed in later, larger studies [15]. Trials of *O. formigenes* demonstrated increased faecal colonization in treatment arms, such as in Hoppe et al. [16], but lactic acid bacteria preparations generally failed to show durable colonization in the study by Goldfarb et al. [12] and Tavasoli et al. [19]. 

Safety and Tolerability 

All interventions were generally well tolerated. Reported adverse events were mild to moderate, mainly gastrointestinal, such as bloating, constipation, and abdominal discomfort. Rare events included renal colic and one case of pyelonephritis [16]. No study reported serious treatment-related adverse outcomes. 

Risk of Bias

Among the RCTs in Table 3, four were double-blind and placebo-controlled, with moderate risk of bias largely due to small sample sizes and incomplete reporting. The non-randomized and pilot studies in Table 4 were assessed as having a high risk of bias using ROBINS-I, primarily due to a lack of controls and blinding. 

**Table 3:** Cochrane risk of bias assessment for randomized controlled trials

Study	Randomization process	Deviations from intended interventions	Missing outcome data	Measurement of the outcome	Selective reporting	Overall risk of bias
Tavasoli et al. [19]	Low risk (randomized, double-blind)	Low risk	Some attrition (dropouts) → Some concern	Low risk (biochemical outcomes, blinded lab)	Low risk	Some concern
Hoppe et al. [16]	Low risk (randomized, placebo-controlled)	Low risk	Minimal missing data	Low risk	Low risk	Low risk
Goldfarb et al. [12]	Low risk (parallel randomized controlled trial)	Low risk	Low risk (all patients analysed)	Low risk	Low risk	Low risk
Jairath et al. [14]	Some concern (details of randomization unclear)	Low risk	Low risk	Some concern (outcome definition incidence-based)	Low risk	Some concern
Lieske et al. [17]	Low risk (randomized, double-blind, stratified by stone history)	Low risk	Low risk (40 enrolled, high completion)	Low risk (standardized diet, blinded biochemical outcomes)	Low risk	Low risk

**Table 4:** ROBINS-I (Risk of Bias in Non-randomized Studies - of Interventions) risk of bias assessment for non-randomized studies

Study	Confounding	Selection of participants	Classification of interventions	Deviations from intended interventions	Missing data	Measurement of outcomes	Selection of reported results	Overall risk of bias
Campieri et al. [11]	Serious (no control group; dietary confounders uncontrolled)	Moderate (selected hyperoxaluric stone formers)	Low	Low	Low	Moderate (biochemical measures objective, but small numbers)	Moderate	Serious
Ferraz et al. [13]	Moderate (diet partially controlled but residual confounding possible)	Moderate (stone formers without hyperoxaluria; convenience sample)	Low	Low	Low	Low (24-h urine objective)	Moderate	Moderate
Hoppe et al. [15]	Serious (mixed renal function; no comparator; baseline differences not adjusted)	Serious (small pilot, highly selected patients)	Low	Low	Low	Low (lab measures robust)	Moderate	Serious
Lieske et al. [18]	Moderate (cross-sectional; observational with some intervention)	Moderate (large but selected cohort of stone formers + controls)	Low	Low	Low	Low (urinary oxalate objective)	Moderate	Moderate

Certainty of Evidence 

A GRADE (Grading of Recommendations Assessment, Development and Evaluation) assessment of the included studies demonstrated that the overall certainty of evidence supporting probiotic or synbiotic interventions for reducing urinary oxalate or preventing kidney stone recurrence is low to very low. Certainty was downgraded due to substantial heterogeneity in study designs, small sample sizes, short intervention durations, and inconsistent reporting of outcomes. Evidence for urinary oxalate reduction was rated as low certainty because effects were inconsistent and frequently imprecise. Evidence for kidney stone recurrence was of very low certainty, as no study was adequately powered or sufficiently long to assess clinical recurrence. Secondary biochemical outcomes and microbiological colonization measures were similarly limited by methodological variability and sparse data. Only the safety profile achieved moderate certainty, with consistent findings that these interventions were well tolerated, and associated primarily with mild gastrointestinal symptoms. Overall, the GRADE assessment indicates that current evidence is insufficient to support probiotic or synbiotic therapy for the clinical prevention of kidney stones. Table 5 summarizes the findings of the GRADE assessment in detail.

**Table 5:** GRADE (Grading of Recommendations Assessment, Development and Evaluation) assessment for included studies RCT: Randomized controlled trial; Ca: Calcium

Outcome	Study types	Overall certainty	Reasons for downgrading
Urinary oxalate excretion	5 RCTs, 4 non-randomized studies	Low	Inconsistency (mixed effects), imprecision (small samples), risk of bias (short duration, incomplete reporting), indirectness (surrogate outcome)
Kidney stone recurrence	Very limited data across studies	Very Low	Serious limitations across all domains: very sparse data, short follow-up, lack of imaging confirmation, heterogeneity
Gut colonization/persistence of administered strains	Small mechanistic sub-studies	Low	Methodological limitations, inconsistent colonization success, high imprecision
Urinary biochemical markers (Ca, citrate, pH, supersaturation)	Reported inconsistently	Very Low	Sparse data, inconsistent reporting, small sample sizes, lack of standardized measurement conditions
Safety and adverse events	All studies	Moderate	Some imprecision due to small sample sizes, but consistent findings showing low risk and mild events

Discussion

This systematic review synthesized evidence from nine clinical studies, including five RCTs and four non-randomized or observational studies, evaluating probiotic and synbiotic interventions targeting oxalate-degrading gut bacteria for the prevention of kidney stones. The primary outcome across studies was urinary oxalate excretion, with kidney stone recurrence rarely reported. 

Overall, the findings suggest that probiotics and synbiotics, including *O. formigenes* preparations and lactic acid bacterial mixtures, have not consistently demonstrated clinically meaningful reductions in urinary oxalate or stone risk. While several pilot studies reported modest decreases in urinary oxalate, these effects were generally not statistically significant, were limited to subgroups, or were not sustained. Only one RCT, Jairath et al. [14], reported a significant reduction in the incidence of hyperoxaluria, but this was compared with an active control, potassium-magnesium citrate, and was not designed to assess stone recurrence. 

Comparison With Existing Literature 

The inconsistency in outcomes may reflect heterogeneity in study design, populations, and interventions. Probiotic efficacy appears highly strain-specific. Hoppe et al. [16] conducted a trial with *O. formigenes* demonstrating successful gut colonization but no consistent biochemical benefit, contrasting with earlier small pilot work by Hoppe et al. [15], which suggested potential reductions in plasma and urinary oxalate. Lactic acid bacteria-based probiotics used by Campieri et al. [11], Goldfarb et al. [12], and Ferraz et al. [13] generally failed to reduce oxalate excretion, though a few individuals experienced marked improvements. This variability highlights the need for a better understanding of host-microbiome interactions, diet, and baseline oxalate metabolism. 

Our findings align with prior narrative reviews, which concluded that evidence for probiotic use in nephrolithiasis prevention remains inconclusive, with most RCTs underpowered to detect meaningful clinical differences. 

Strengths and limitations of the evidence

The strengths of the available evidence include several double-blind, placebo-controlled RCTs with objective biochemical endpoints; however, important limitations remain. Most trials enrolled fewer than 50 participants per arm and were of short duration, typically ≤8 weeks, which is insufficient to assess stone recurrence. Considerable heterogeneity was also present, with interventions varying in probiotic strains, dosages, and delivery vehicles, making comparisons challenging. Outcomes were inconsistently reported, with some studies focusing on continuous urinary oxalate excretion, others on the incidence of hyperoxaluria, and a few assessing imaging-confirmed recurrence. Furthermore, many non-randomized studies and some RCTs were judged to have a high or unclear risk of bias. Collectively, these issues reduce the overall certainty of the evidence. 

Clinical and research implications 

At present, routine clinical use of probiotics or synbiotics for kidney stone prevention cannot be recommended based on available evidence. The consistent absence of significant effects in high-quality RCTs underscores the challenges in translating microbiome-targeted therapies into clinically meaningful outcomes.

Future research should prioritize larger, adequately powered, multicentre RCTs with longer follow-up periods to assess kidney stone recurrence, rather than focusing solely on biochemical surrogates. Strain-specific evaluations, particularly of *O. formigenes* and engineered microbial consortia, are needed, ideally using standardized dosing regimens to enhance comparability across studies. Trials should also integrate dietary and metabolic factors, given that diet strongly influences oxalate handling and may confound probiotic effects. In addition, mechanistic endpoints, such as microbiome sequencing and assessments of colonization durability, will be essential to clarify host-microbiome interactions and better understand how these interventions might reduce stone risk. 

## Conclusions

This systematic review demonstrates that, although probiotics and synbiotics targeting oxalate-degrading bacteria are safe and biologically plausible interventions, current evidence does not support their effectiveness in reducing urinary oxalate or preventing kidney stone recurrence. Further well-designed RCTs are required before these therapies can be considered a reliable preventive strategy for nephrolithiasis. 

## Appendices

Appendix A

**Table 6:** Search strategies for various databases

Search strategy	Database	Number of papers filtered
("Kidney Calculi"[Mesh] OR "Urolithiasis"[Mesh] OR "Nephrolithiasis"[Mesh] OR "kidney stone*"[tiab] OR "renal stone*"[tiab] OR "urinary stone*"[tiab] OR urolithiasis[tiab] OR nephrolithiasis[tiab]) AND ("Probiotics"[Mesh] OR "Synbiotics"[Mesh] OR "Prebiotics"[Mesh] OR probiotic*[tiab] OR synbiotic*[tiab] OR prebiotic*[tiab]) AND ("Oxalobacter formigenes"[tiab] OR "Oxalobacter"[tiab] OR "Lactobacillus"[tiab] OR "Bifidobacterium"[tiab] OR "oxalate-degrading bacteria"[tiab] OR "oxalate degradation"[tiab])	PubMed / PMC / Medline	52
(TITLE-ABS-KEY("kidney stone*" OR "renal stone*" OR "urinary stone*" OR urolithiasis OR nephrolithiasis)) AND (TITLE-ABS-KEY(probiotic* OR synbiotic* OR prebiotic*)) AND (TITLE-ABS-KEY("Oxalobacter formigenes" OR Oxalobacter OR Lactobacillus OR Bifidobacterium OR "oxalate-degrading bacteria" OR "oxalate degradation")) AND PUBYEAR > 2014 AND PUBYEAR < 2026	Scopus	115
1. 'urolithiasis'/exp OR 'nephrolithiasis'/exp OR 'kidney calculus'/exp; 2. (kidney stone* OR renal stone* OR urinary stone* OR urolithiasis OR nephrolithiasis).ti,ab,kw.; 3. 1 OR 2; 4. 'probiotic agent'/exp OR 'synbiotic agent'/exp OR 'prebiotic agent'/exp; 5. (probiotic* OR synbiotic* OR prebiotic*).ti,ab,kw.; 6. 4 OR 5; 7. 'oxalobacter formigenes'/exp OR 'oxalobacter'/exp OR 'lactobacillus'/exp OR 'bifidobacterium'/exp; 8. ("Oxalobacter formigenes" OR Oxalobacter OR Lactobacillus OR Bifidobacterium OR "oxalate-degrading bacteria" OR "oxalate degradation").ti,ab,kw.; 9. 7 OR 8; 10. 3 AND 6 AND 9; 11. Limit 10 to yr="2015 - 2025"	EMBASE	65
#1 (kidney stone* OR renal stone* OR urinary stone* OR urolithiasis OR nephrolithiasis):ti,ab,kw; #2 (probiotic* OR synbiotic* OR prebiotic*):ti,ab,kw; #3 ("Oxalobacter formigenes" OR Oxalobacter OR Lactobacillus OR Bifidobacterium OR "oxalate-degrading bacteria" OR "oxalate degradation"):ti,ab,kw; #4 #1 AND #2 AND #3	Cochrane Library	12
